# Detection of Mycobacterium tuberculosis Complex Using the Xpert MTB/RIF Ultra Assay on the Stool of Pediatric Patients in Dushanbe, Tajikistan

**DOI:** 10.1128/spectrum.03698-22

**Published:** 2023-01-09

**Authors:** Michael L. Rekart, Lidiya Mun, Aung Aung, Diana Gomez, Winston Mulanda, Jarmila Kliescikova, Norman Sitali, Asliddin Rajabov, Shahnoza Azamova, Bobojon Pirmahmadzoda, Jay Achar, Jose Luis Alvarez, Jane Greig, Philipp du Cros, Animesh Sinha

**Affiliations:** a Médecins sans Frontières, Amsterdam, The Netherlands; b Médecins sans Frontières, Dushanbe, Tajikistan; c National Tuberculosis Programme, Dushanbe, Tajikistan; d Dushanbe City TB Center, Dushanbe, Tajikistan; e Department of Global Public Health, Karolinska Institutet, Stockholm, Sweden; f Médecins sans Frontières, London, United Kingdom; g Burnet Institute, Melbourne, Victoria, Australia; AP-HP

**Keywords:** *Mycobacterium tuberculosis*, Xpert MTB/Rif Ultra, pediatric infectious disease, stool

## Abstract

We report the findings of a prospective laboratory diagnostic accuracy study to evaluate the sensitivity, specificity, and predictive values of the Xpert MTB/RIF Ultra assay for Mycobacterium tuberculosis detection in fresh stool specimens from children under 15 years of age with confirmed tuberculosis (TB) disease from Dushanbe, Tajikistan. Six hundred eighty-eight (688) participants were enrolled from April 2019 to October 2021. We identified 16 participants (2.3%) with confirmed TB disease, defined as ≥1 TB sign/symptom plus microbiologic confirmation. With the Xpert MTB/RIF Ultra assay for stool, we found a sensitivity of 68.8% (95% CI, 46.0 to 91.5) and a specificity of 98.7% (95% CI, 97.8 to 99.5) in confirmed TB disease. Our results are comparable to other published studies; however, our cohort was larger and our confirmed TB disease rate lower than most. We also demonstrated that this assay was feasible to implement in a centralized hospital laboratory in a low-middle-income Central Asian country. However, we encountered obstacles such as lack of staffing, material ruptures, outdated government protocols, and decreased case presentation due to COVID-19. We found eight patients whose only positive test was an Xpert Ultra stool assay. None needed treatment during the study; however, three were treated later, suggesting such cases require close observation. Our report is the first from Central Asia and one of a few from a low-middle-income country. We believe our study demonstrates the generalizability of the Xpert MTB/RIF Ultra assay on fresh stool specimens from children and provides further evidence supporting WHO’s approval of this diagnostic strategy.

**IMPORTANCE** The importance of this report is that it provides further support for WHO’s recent recommendation that fresh stool is an acceptable sample for GeneXpert TB testing in children, especially small children who often cannot produce an adequate sputum sample. Diagnosing TB in this age group is difficult, and many cases are missed, leading to unacceptable rates of TB illness and death. In our large cohort of children from Dushanbe, Tajikistan, the GeneXpert stool test was positive in 69% of proven cases of TB, and there were very few false-positive tests. We also showed that this diagnostic strategy was feasible to implement in a low-middle-income country with an inefficient health care delivery system. We hope that many more programs will adopt this form of diagnosing TB in children.

## INTRODUCTION

The World Health Organization (WHO) estimates that 9.9 million people developed active tuberculosis (TB) disease in 2020. Eleven percent (11%) are estimated to have occurred in children under 15 years of age (1). A mathematical modeling study from 2017 estimated an annual mortality of around 239,000 cases in this age group (2). Eighty percent (80%) of pediatric deaths occurred in children under 5 years of age. TB is a top-ten cause of mortality in children globally. In 2019, WHO estimated that 490,000 people developed multidrug/rifampicin-resistant TB (MDR/RR-TB). Tajikistan has been designated a high-burden MDR/RR-TB country by WHO (3), with approximately 7,700 cases estimated in 2019. Around 200 of these were children, and 73% of these children were not notified (4). TB can progress rapidly in children because of their immature immune system, making diagnosis and treatment more urgent. Prompt detection of TB in children is especially important to enable more timely treatment and improved outcomes (5, 6).

Children with pulmonary TB often present with atypical signs and symptoms, especially HIV-positive or malnourished children (5). Clinically, pulmonary TB in younger children presents with pauci-bacillary, noncavitary pulmonary disease, making microbiological confirmation difficult. Obtaining sputum samples from young children is challenging due to lack of sufficient tussive force to produce adequate samples by expectoration alone. They usually swallow their sputum when they cough. For this reason, an adequate sample can only be obtained by nasogastric aspiration or sputum induction in most cases. In 2013, Médecins sans Frontières (MSF) supported the Tajikistan Ministry of Health (MOH) in implementing sputum induction as a diagnostic procedure to obtain clinical samples from children for bacteriological confirmation. Sputum induction and nasogastric aspiration are invasive procedures that require specialized equipment and skills often lacking at the primary health care level. Because TB in young children is often pauci-bacillary, diagnostic methods with high sensitivity are necessary to maximize the likelihood of detection of Mycobacterium tuberculosis (MTB) bacilli. A microbiological diagnosis reduces the risk of misdiagnosis, especially for drug-resistant TB, and the risk of delayed initiation of effective treatment (6).

Since the release and rollout of the Xpert MTB/RIF assay and later the Xpert MTB/RIF Ultra assay, this fast and sensitive technology has dramatically impacted case detection using sputum, and ultimately treatment outcomes (7). Recently, WHO has recommended stool for the diagnosis of TB in children using these technologies (8). Stool is easy to collect, and studies have demonstrated that the molecular cartridge-based Xpert MTB/RIF assay (Cepheid, Sunnyvale, CA, US) or the more sensitive Xpert MTB/RIF Ultra assay can reliably detect MTB in stool (8 to 11). GeneXpert instruments are widely available at the secondary care level, and availability at the primary care level is rapidly expanding in most countries, as part of the End TB strategy (12, 13). The application of Xpert testing using stool provides an opportunity for sensitive and noninvasive diagnosis of TB in children near the point of care.

There are different methods of stool processing, some requiring centrifugation and buffer preparation (9). The simple one-step stool (SOS) processing method adds stool directly to the sample reagent provided in the Xpert kit to release the bacteria from the feces and to inactivate the M. tuberculosis bacilli. Sedimentation by gravity follows, assuming the bacilli will float to the top in the watery solution due to their lipid-containing cell wall (9, 14).

In 2019, MSF with support from the Tajikistan National TB Program (NTP) initiated a study to evaluate the use of the Xpert MTB/RIF Ultra assay on stool from children at high risk for TB.

## RESULTS

**FIG 1:**
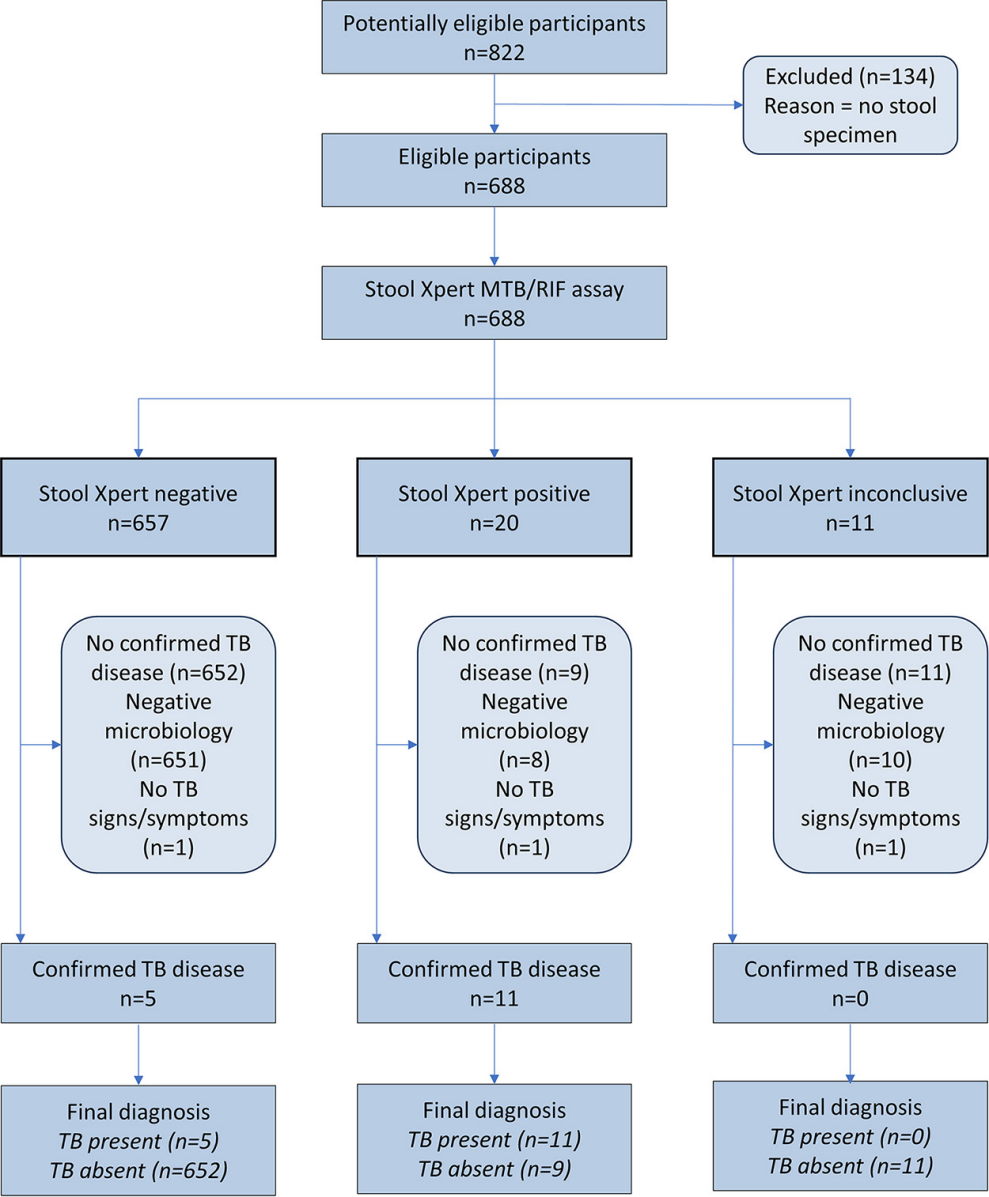
Flowchart of participants through the study.

Fig. 1 consists of a STARD (Standards for Reporting Diagnostic Accuracy Studies) diagram to report the flow of participants through the study.

In the 31 months from April 2019 to October 2021, we enrolled 822 subjects from our sample size target of 1,430 subjects over 22 months. Because of inadequate enrollment within the context of ongoing uncertainty due to the COVID-19 pandemic, the study was terminated early.

Of the 822 patients enrolled, 134 (16.3%) did not provide a stool sample. Among this group, 75 (56.0%) were female, 59 (44.0%) male, 71 (53.0%) 1 to 4 years, 39 (29.1%) 5 to 9 years, 23 (17.2%) 10 to 14 years, and one >14 years. The baseline characteristics for the remaining 688 participants are shown in Table 1.

**TABLE 1:** Baseline characteristics

Baseline characteristic	No.	% of total
Gender		
Male	304	44.2
Female	384	55.8
Age at enrolment		
0 to 4 yrs	299	43.5
5 to 9 yrs	292	42.4
10 to 14 yrs	95	13.8
>14 yrs	2	0.3
History of TB contact		
Household	292	42.4
Casual	22	3.2
Other, e.g., teacher, kindergarten, hospital	39	5.7
Unknown	66	9.5
None stated	269	39.1
TB signs/symptoms		
Cough >2 wks only	276	40.1
Unexplained fever >1 wk only	8	1.2
Wt loss/poor growth >3 mo only	13	1.9
Cough >2 wks + unexplained fever >1 wk	72	10.5
Cough >2 wks + wt loss/poor growth >3 mo	0	0
Unexplained fever >1 wk + wt loss/poor growth >3 mo	109	15.8
Cough >2 wks + unexplained fever >1 wk + wt loss/poor growth >3 mo	114	16.6
Pneumonia, unexplained hepatosplenomegaly, sepsis-like fever in <1-yr-old children	1	0.1
Blank, information missing	2	0.3
No signs/symptoms	93	13.5
Total with ≥1 sign/symptom	593	86.2
Total with cough >2 wks	462	67.2
Total with unexplained fever >1 wk	303	44.0
Total with wt loss/poor growth >3 mo	236	34.3
History of previous TB treatment		
First-line TB treatment (cured/completed 5, failed 1, lost to follow-up 1)	7	1.0
No previous TB treatment	282	41.0
Unknown	399	58.0
Chest X-ray results		
Normal	250	36.3
Abnormal	333	48.4
Unknown, not done	105	15.3
TB skin test (TST) results		
Positive	135	19.6
Negative	283	41.1
Unknown, not done	270	39.2
HIV test results		
Positive	9	1.3
Negative	185	26.9
Unknown, not done	494	71.8

The guardians of 34 patients refused participation, mostly because they lived far away. Their baseline characteristics were as follows: female 19 (55.9%), male 15 (44.1%), age 0 to 4 years 14 (41.2%), 5 to 9 years 9 (26.5%), and 10 to 14 years 11 (32.3%).

Slightly more than half had a history of TB contact, most of which (292) were household contacts, and 86.2% reported one or more signs/symptoms. In the 93 patients with no signs/symptoms, 61 had a history of TB contact and an additional 11 had an abnormal chest X ray. These 93 plus the two patients with no information on signs/symptoms were classified as no TB disease. Almost half had an abnormal chest X ray with abnormalities, including lymphadenopathy 31, disseminated disease 4, fibrosis 2, fibro-cavernous disease 4, infiltration 15, and “other” 18.

For HIV, there were only nine positive tests (1.3%). In the 16 MTB confirmed cases, the HIV test was positive for one patient. That patient had a positive Xpert sputum, a positive MGIT and LJ result, and a positive Xpert stool result.

There were 18 Xpert MTB/RIF Ultra positive sputum samples (2.8%) and 670 negative samples.

Three patients with a positive sputum Xpert result were asymptomatic and therefore not classified as confirmed TB disease according to our criteria. These three cases had the following findings:

Case number 1: Household contact, no information on CXR or TST or previous treatment, invalid stool Xpert, negative culture, no Hain test.
Case number 2: Household contact, normal CXR, positive TST, no information on previous treatment, negative stool Xpert, negative culture, no Hain test.
Case number 3: No history of TB contact, abnormal CXR, negative TST, no previous treatment, positive stool Xpert, negative culture, no HAIN test.

The remaining 15 cases were classified as confirmed TB disease (see Table 2). There was one additional confirmed TB disease case that had signs/symptoms, a positive MGIT, and negative Xpert Ultra sputum and stool assays. Thus, 16 (15) participants had confirmed TB disease (2.3%) (Table 2). All 16 patients were documented to be on treatment within 6 weeks.

**TABLE 2:** Findings and characteristics of the 16 confirmed TB disease^*a*^ cases

Characteristic	(+)^*b*^ sputum Xpert only (*n* = 6)	(+) sputum Xpert and (+) TB culture (*n* = 9)	(+) TB culture only (*n* = 1)	Total (*n* = 16) (% total)
Male	4	5	0	9 (65.2%)
Female	2	4	1	7 (43.8%)
0 to 4 yrs of age	2	4	1	7 (43.8%)
5 to 9 yrs of age	3	1	0	4 (25.0%)
10 to 14 yrs of age	0	4	0	4 (25.0%)
>14 yrs of age	1	0	0	1 (6.2%)
History of TB contact	5	5	0	10 (62.5%)
History of previous TB treatment	0	0	0	0 (0.0%)
Cough >2 wks	5	6	1	12 (75.0%)
Fever >1 wk	3	7	1	11 (68.8%)
Wt loss/poor growth >3 mo	3	7	1	11 (68.8%)
Abnormal chest X-ray	5	4	0	9 (65.2%)
(+) TB skin test	2	2	0	4 (25.0%)
(+) HIV test	0	1	0	1 (6.2%)
(+) Stool Xpert	3	8	0	11 (68.8%)

a Confirmed TB disease ≥1 TB sign/symptom + microbiologic confirmation.

b (+), positive.

The MGIT culture was positive in 10 patients, negative in 302, contaminated in 3 patients, and not done in 373. The Hain MDRTBplus was performed on sputum for only 25 cases with one positive result. This case had a positive result on culture and sputum Xpert and stool Xpert assays. This strain was susceptible to rifampicin and INH.

There were 190 cases (27.6%) that met the study definition of probable TB disease; 286 cases (41.6%) that met the definition for possible TB disease; and 196 (28.5%) cases of no TB disease. We did not systematically collect information on alternative diagnoses in participants without confirmed TB disease.

**FIG 2:**
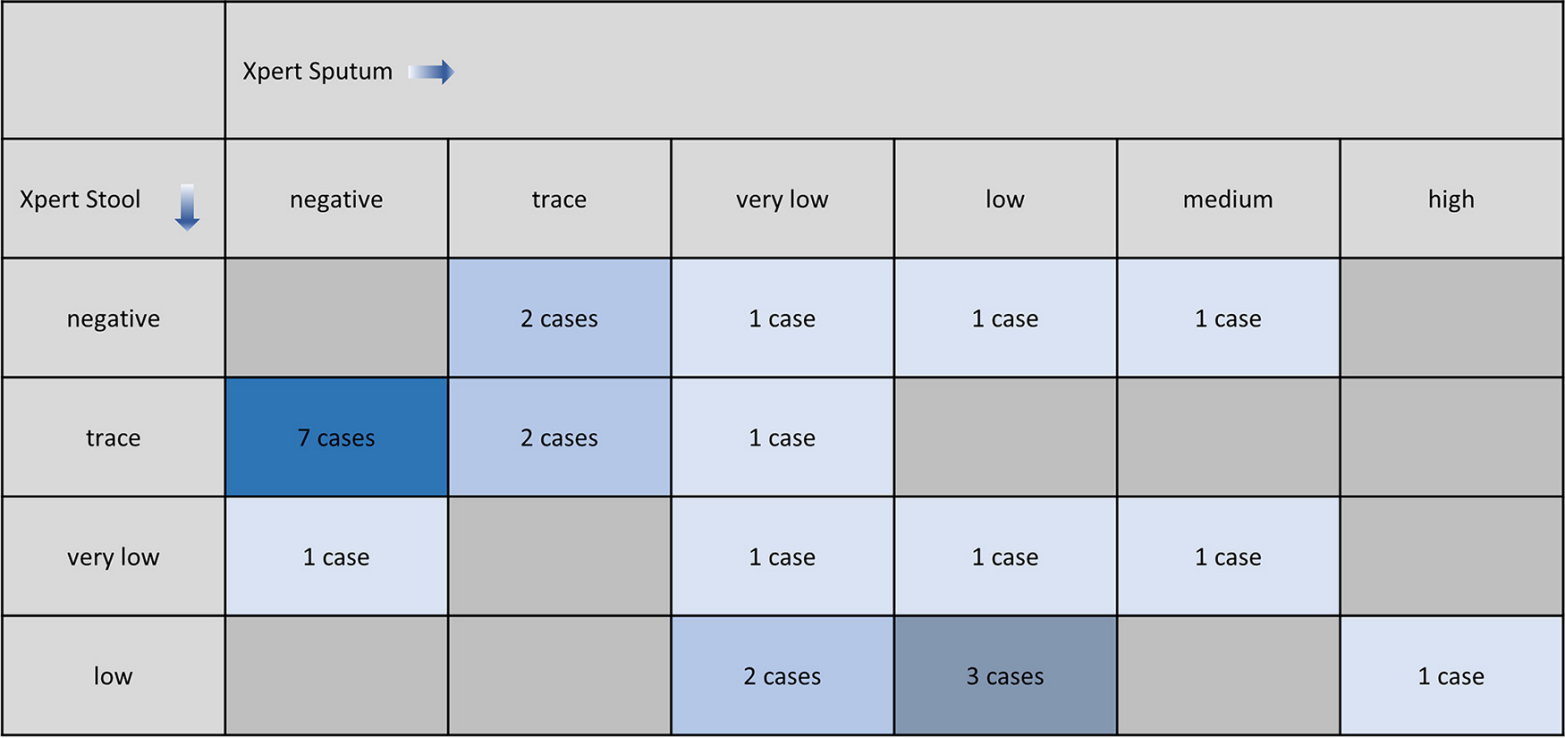
Correlation of semiquantitative results for Xpert MTB/RIF Ultra on sputum and stool (includes 2 patients without confirmed TB disease because of no TB signs/symptoms).

Semiquantitative categories of the Xpert MTB/RIF Ultra assay on sputum and stool are compared in Table 3, and the correlation of the semiquantitative results for Xpert MTB/RIF Ultra between sputum and stool are shown in Fig. 2. For stool, 20 of 688 (2.9%) were positive by Xpert MTB/RIF Ultra. Of these, 10 (50.0%) had trace results. Additionally, 10 had an invalid result. All 10 tests with an invalid result were repeated a second time with the same reading.

**TABLE 3:** Semiquantitative categories of Xpert MTB/RIF Ultra assay on sputum and stool

Xpert MTB/RIF Ultra assay	Induced sputum (688)	Stool specimen (688)
Total positive	18 (2.6%)	20 (2.9%)
High	1	0
Medium	2	0
Low	6	6
Very low	5	4
Trace call	4	10

We found 10 trace results on stool and 4 on sputum. Two samples were trace on both. One of these was MGIT negative with no TB signs/symptoms. The other was confirmed TB disease with a positive MGIT and LJ, as well as TB signs/symptoms. For the other two trace results on sputum, the stool Xpert and MGIT were negative but the patients had TB signs/symptoms and were therefore confirmed TB disease. Of the remaining six cases of trace results on Xpert stool that were Xpert sputum negative, none were MGIT/LJ positive or confirmed TB disease. There were 10 invalid stool Xpert tests.

The sensitivity, specificity, positive predictive value, and negative predictive value for Xpert MTB/RIF Ultra stool testing in confirmed TB disease are shown in Table 4. The sensitivity of Xpert MTB/RIF Ultra on stool was 68.8%, specificity 98.7%, positive predictive value 55.0%, and negative predictive value 99.3%.

**TABLE 4:** Performance of the Xpert MTB/Rif Ultra assay for fresh stool in pediatric TB

Patient group	Xpert MTB/RIF Ultra on stool
Reference standard	Confirmed TB disease (>1 sign/symptom + microbiologic confirmation)
Sensitivity	68.8% (95% CI, 46.0 to 91.5) (11/16)
Specificity	98.7% (95% CI,97.8 to 99.5) (663/672)
Positive predictive value (PPV)	55.0% (95% CI,33.2 to 76.8) (11/20)
Negative predictive value (NPV)	99.3% (95% CI,98.6 to 99.9) (663/668)

The agreement between Xpert MTB/RIF Ultra stool and induced sputum was moderate when used for all enrollees (kappa value = 0.522). There were eight subjects whose only positive test was an Xpert stool. None was started on TB treatment at the time of the positive stool test. In telephone follow-up after 6 months, three were subsequently commenced on TB treatment.

One patient was identified as Xpert MTB/RIF Ultra rifampicin resistant on a stool specimen but was not confirmed TB disease. This patient was not put on RR-TB treatment because their Xpert MTB/RIF Ultra sputum test did not show rifampicin resistance. Overall, 21 patients were treated for RR-TB based on the decision of the Tajikistan TB Consilium of experts because of a history of contact with an RR-TB patient. None of these cases had an Xpert MTB/RIF positive sputum or resistance demonstrated by DST.

There were no serious adverse events reported from performing any of the diagnostic tests included in this study.

## DISCUSSION

The key result from our study was the sensitivity of 68.8% (95% CI, 46.0 to 91.5) for the Xpert MTB/RIF Ultra assay on fresh stool samples from children with confirmed TB disease and the corresponding specificity of 98.7% (95% CI, 97.8 to 99.5). Our results are consistent with the literature, with a systematic review by WHO finding an overall sensitivity of 53% in children 0 to 9 years of age and an overall specificity of 98% (8). A second systematic review and meta-analysis by Gebre et al. (15) found a pooled sensitivity for Xpert MTB/RIF Ultra on stool of 0.50 (95% CI, 0.44 to 0.56) compared with bacteriologically confirmed TB on respiratory specimens. The sensitivities reported across included studies ranged from 32% to 85%. The pooled specificity was 0.99 (95% CI, 0.98 to 0.99).

We found a higher frequency of invalid test results from stool samples than induced sputum. One reason may be the presence of PCR inhibitors in stool samples; the other reason may be that debris can lead to clotting and errors in the test run (14, 16).

Our protocol specified culture only for Xpert MTB/RIF Ultra sputum positive cases, because of limited national capacity, but we were able to obtain cultures on 298 Xpert MTB/RIF Ultra sputum negative cases. This was fortunate because one case with a negative Xpert MTB/RIF Ultra sputum was confirmed TB disease because of a positive MGIT.

Our reference standard for confirmed TB disease (≥1 TB sign/symptom plus microbiologic confirmation) was defined in January 2019 when the concept of “minimal” or “subclinical” TB disease requiring treatment was less clear and accepted. If we had used only microbiologic confirmation, we would have had an additional 3 cases confirmed by Xpert sputum, from which there was one positive, one negative, and one invalid Xpert stool result. This would have resulted in a slightly lower sensitivity of 63.2% (12 of 19) for the Xpert MTB/RIF Ultra assay on stool.

We demonstrated the feasibility of performing Xpert MTB/RIF Ultra on stool specimens using a standardized laboratory protocol in Dushanbe, Tajikistan. We were able to adapt our protocol to the standard laboratory procedures in the PD Hospital with only 10 invalid results out of 688 tests (1.5%). This was more than for Xpert sputum testing (0 invalid tests) but acceptable for a newly introduced test, compared with other studies. Nine of 10 invalid results occurred in the first year of the study, suggesting early issues with the test performance technique. There was no need for additional human resources or significant extra training. We did not receive any stool samples that were insufficient or unusable. The declining enrollment in the second and third years of our study were due to factors extraneous to the lab, including inadequate staffing, ruptures of materials, inflexible and outdated government standards, and decreased attendance due to COVID-19.

Although not part of our protocol, we tried to follow up cases whose only positive test was the Xpert MTB/RIF Ultra stool test to see whether they were subsequently diagnosed or treated for TB. At 6 months after study conclusion, we found that three of eight such cases were later treated for TB disease. The other five cases were not treated for TB, or no information was obtainable. Our results suggest that this group needs close follow-up.

One of the study strengths was that it was a prospective study with a large number of enrolled patients with both induced sputum and stool testing.

The limitations of our study included inadequate recruitment overall requiring early termination of the study because of declining recruitment over time due to human resource issues, changing staff, fear of attending health care facilities because of COVID-19, and ruptures involving Xpert cartridges, reagents, and other supplies. We also observed a lower-than-expected prevalence of confirmed TB disease (actual 2.5% versus predicted 5.0%), resulting in wider 95% confidence intervals. Missing data, exclusion due to lack of stool specimen, and lack of MGIT for all included were also limitations. Finally, we have HIV test results on only 28.2% of participants.

### Conclusions and recommendations.

The findings of our study support the use of the Xpert MTB/RIF Ultra assay on fresh stool specimens from children as part of the diagnostic armamentarium for pediatric TB. Our results also show that the modified Banada method of sample preparation is feasible and may lead to a relatively high sensitivity rate.

## MATERIALS AND METHODS

This was a prospective laboratory diagnostic accuracy study conducted in a single laboratory in Dushanbe, Tajikistan, between April 2019 and October 2021. Recruitment, follow-up, and data collection for the study occurred during this period. We did selective follow-up on participants whose clinical status was uncertain.

This study was conducted at the Dushanbe Pediatric Department (PD) Hospital, a referral center for pediatric TB and sputum-induction services. Over 1,000 sputum-induction procedures are performed annually by six trained nurses. Specimens are tested using Xpert MTB/RIF assay (Cepheid, Sunnyvale, CA, USA) in an on-site laboratory by two trained laboratory technicians. On average, 65 children under 15 years of age are referred for diagnostic sputum induction each month, all of whom were eligible for inclusion in this study. Around 6.5% of sputum induction samples are Xpert MTB/RIF Ultra positive at this facility. All sputa for this study were induced.

The study was conducted by a medical team of nurses and laboratory technicians working on site. Data collection and analysis were supported by a data entry operator. A laboratory manager and nurse supervisor were responsible for training, implementation, and monitoring, including the drafting of regular monthly progress reports. They were also responsible for ensuring good collaboration between MSF and MOH staff working in the laboratory and sputum induction facilities. Staff nurses ensured that information was given to patients/guardians and that informed consent was obtained prior to sample collection. All data were recorded on standardized forms, and samples were collected according to standard operation procedures (SOPs). Laboratory technicians at the site were responsible for sample processing and analysis according to study SOPs, recording results on designated forms and databases and giving results to the responsible doctors in a timely manner.

### Inclusion criteria.

A consecutive series of children under 15 years of age who presented or were referred with suspected pulmonary TB to the Pediatric Department Hospital in Dushanbe were eligible for inclusion. Most of these children had symptoms or signs suggesting TB, but some were asymptomatic contacts of TB cases or had abnormal chest X rays.

### Exclusion criteria.

Exclusion criteria were the following: children who received >24 hours of TB treatment (excluding isoniazid prophylaxis therapy) within the preceding 30 days; refusal to participate in the study; parent/guardian unable to give informed consent; or collection of a sputum and stool sample within 3 days of each other not expected to be possible. Liquid stool was accepted for processing, and the presence of diarrhea was not an exclusion criterion.

### Sample collection and processing.

We asked parents/guardians to collect a stool sample after the routine collection of an induced sputum specimen from each subject. A sterile stool container and instructions were provided for specimen collection and handling. Once collected, the parent/guardian was asked to refrigerate the sample and bring it to the laboratory within 12 h to be stored at the recommended temperature for fresh stool samples of 2 to 8°C. If no refrigerator was available at home, sample collection was done in the morning of the day the parent/guardian visited the clinic. Following delivery to the laboratory, the stool specimen was kept in a cool and dry place and processed within 2 h. Stool samples were frozen between −20°C and −15°C if processing could not occur immediately.

As per local policy, two sputum specimens were collected, one for molecular testing and a second for mycobacterial culture. Sputum specimens were collected at the clinic and sent to the laboratory, where they were kept between 2 and 8°C until processing. Processing was done within 24 h where possible and not more than 72 h after collection.

We attempted to collect stool and sputum samples within 3 days of each other. Patients not able to produce the requested samples were asked to make further attempts during the following days, but not after 1 week. Only patients able to submit at least one sputum specimen and one stool sample within 3 days of each other were included in the study. Samples did not require additional storage measures for the purposes of the study, and they were discarded per local policy.

Sputum samples were processed using the Xpert MTB/RIF Ultra cartridge and standard smear microscopy in the Dushanbe TB PD Hospital Laboratory. All Xpert MTB-detected specimens were referred to the National Reference Laboratory for culture and phenotypic drug susceptibility testing (DST).

The slightly modified “Banada” method (16) that uses less quantity of processed stool was used for this study. For stool samples, 0.6 g (±0.2 g) were weighed and processed according to the study protocol as follows: two milliliters (mL) of stool processing buffer (SPB), containing AL buffer (Qiagen, Valencia, CA) and 10% Poly-vinyl-pyrrolidone (Sigma-Aldrich, St. Louis, MO), plus 2 mL of Xpert MTB/RIF sample reagent (SR) (Cepheid), and approximately 10 3-mm glass beads (Fisher Scientific, Pittsburgh, PA), were added to the stool. After a short mix and 30 min of incubation, the mixture was passed through a syringe filter (fitted with glass wool to capture the stool debris) into a clean collection vial. Two mL of this filtrate was loaded into the sample chamber of an Xpert MTB/RIF Ultra assay cartridge. Centrifugation was not used. Subsequent sample processing and PCR were performed in accordance with the manufacturer's recommendations using the GeneXpert instrument. The analysis was done by GxDx software version 4.8.

### Data collection.

Data collection utilized standardized forms. Participant data were collected at the time of study enrollment. Laboratory data were retrieved following Xpert MTB/RIF Ultra testing of specimens. End-of-study follow-up data were collected from centralized TB registers and family clinic records. All hard copies of data were securely stored in the PD Hospital Laboratory, and all computerized information was password-protected. Data were recorded in a dedicated Excel database. Data were backed up regularly and stored by the in-country data entry supervisor and the primary investigator. Access to study data were restricted to investigators involved in data management and analysis.

### Study endpoints.

The study’s primary endpoint was the result of stool Xpert MTB/RIF Ultra assay from children with confirmed TB disease, as defined in Table 5. Confirmed TB disease served as the reference standard for the determination of sensitivity, specificity, and positive and negative predictive values.[Table tab5]

**TABLE 5 tab5:** Classification of certainty of the diagnosis of TB in children

Category	Definition
Confirmed TB disease	At least 1 sign or symptom of TB disease, including cough >2 wks, unexplained fever >1 wk, and poor growth or wt loss over the preceding 3 months^*a*^ and microbiological confirmation of M. tuberculosis in sputum
Probable TB disease	At least 1 sign or symptom of TB disease and a chest X ray consistent with intrathoracic disease and presence of one of the following: (i) clinical response to TB treatment; (ii) exposure to a source case with TB disease; (iii) immunological evidence of TB infection.
Possible TB disease	At least 1 sign or symptom of TB disease and either a chest X ray consistent with intrathoracic disease or presence of one of the following: (i) clinical response to TB treatment; (ii) exposure to a source case with TB disease; (iii) immunological evidence of TB infection.
No TB disease	Patients that did not fall into the above categories.

a For children <1 year old, the following signs were also accepted: pneumonia, unexplained hepatosplenomegaly, and sepsis-like illness.

Stool Xpert MTB/RIF Ultra results were classified as follows: MTB detected in stool (high, medium, low, or very low); MTB detected in stool (trace); MTB not detected in stool; or invalid, error, unknown, or no result recorded.

### Case definitions.

Participant risk of TB was classified based on consensus definitions using clinical, radiological, and laboratory assessments (Table 5) (13).

Microbiological confirmation of TB disease constituted one or more of the following results from sputum specimens: positive smear microscopy results; MTB detected by Xpert MTB/RIF or Xpert MTB/RIF Ultra; MTB complex detected by Hain MTBDRplus; MTB complex detected in MGIT or LJ culture. Microbiological confirmation of rifampicin resistance (RR) constituted the detection of RR from respiratory specimens by Xpert MTB/RIF or Xpert MTB/RIF Ultra, Hain MTBDRplus, or phenotypic DST from MGIT or LJ.

### Statistical analysis.

For estimating sample size, we hypothesized that the sensitivity of Xpert MTB/RIF Ultra testing on stool specimens would be >60% in children under 15 years of age with confirmed TB disease.

All other individuals that did not meet our reference standard were considered not confirmed TB, including those with probable TB, possible TB, and no TB.

Sensitivity, specificity, negative predictive value, and positive predictive value were calculated by defining true or false positives and true or false negatives against the reference standard of confirmed TB disease. Confidence intervals were calculated using the simple asymptotic method based on the normal approximation to the binomial distribution. The proportion testing indeterminate is reported. Categorical variables are summarized as counts and percentages. Following repetition in the laboratory, repeat invalid sputum and stool tests (e.g., Xpert Ultra error) were excluded from the primary analysis. Statistical analysis was performed using Stata 14 (Stata Corporation, College Station, TX, USA) and R (R Foundation for Statistical Computing, Vienna, Austria).

Our study was implemented through a partnership between the Tajikistan MOH and MSF Holland. The Tajikistan National TB Control Program and the MOH actively participated in protocol development and supported study implementation. MSF acted as the study sponsor and provided funding for resources that were not part of the base budget of the MOH.

### Ethical considerations, permits, and authorship.

The Tajikistan National TB Control Program and the MOH granted permissions for the study to take place, actively participated in protocol development, and supported study implementation. The study protocol was approved by the Ethical Review Board of MSF on 18 January 2019, approval number 18103.

We obtained consent from the parents or guardians of minors included in the study. Children deemed competent were asked to sign the written consent alongside their guardian or parent. Those who had sufficient understanding were asked to give their assent according to local practice.
